# Elementary Microscopical Technology: A Manual for Students

**Published:** 1887-06

**Authors:** 


ELEMENTARY MICROSCOPICAL TECHNOLOGY. A MANUAL FOR STUDENTS OF MICROSCOPY. IN THREE PARTS. PART I, *By* FRANK L. JAMES, PH. D., M.D., *Illustrated, pp. 107.* ST. LOUIS MEDICAL AND SURGICAL JOURNAL COMPANY, 1887.

The author has endeavored to make his description of the steps of preparing tissue and mounting a slide so complete that a beginner using this book, will require no other information. It attempts nothing more.

It contains many good suggestions but deals largely with biological microscopical technology, and is far less useful for medical students than other books previously published. The literary quality of the book can scarcely be commended.

